# Association between serum Klotho levels and hypothyroidism in older adults: NHANES 2007–2012

**DOI:** 10.1038/s41598-024-62297-4

**Published:** 2024-05-20

**Authors:** Yan Wang, Ben Hu, Suyun Yang

**Affiliations:** 1 Academy of Medical Sciences, Shanxi Medical University, Taiyuan, 030001 Shanxi Province China; 2 Department of Nuclear Medicine, First Hospital of Shanxi Medical University, Taiyuan, 030001 Shanxi Province China; 3 Department of Cardiology, The Second People’s Hospital of Hefei, Hefei Hospital Affiliated to Anhui Medical University, Hefei, 230011 Anhui China

**Keywords:** Biomarkers, Diseases, Population screening, Medical research, Biomarkers, Endocrine system and metabolic diseases

## Abstract

Whether Klotho plays any role in hypothyroidism is unknown. This study aimed to determine the relationship between serum Klotho levels and hypothyroidism in older adults. From the 2007 to 2012 National Health and Nutrition Examination Survey (NHANES), 1444 older adults aged 65–79 were included in this cross-sectional study. Hypothyroidism was diagnosed using participants' reports of current medications and TSH tests. Klotho was measured using an enzyme-linked immunosorbent assay. The relationship between serum Klotho levels and hypothyroidism in older people was analyzed by one-way analysis of variance, multiple linear regression models, subgroup analyses, interaction tests, smoothed curve fitting, and threshold effects. A total of 209 (14.47%) participants were identified as having hypothyroidism. Serum Klotho (ln transformation) is independently and significantly negatively associated with the risk of hypothyroidism after complete adjustment for confounders (OR = 0.49, 95% CI 0.31–0.80; P = 0.0039). The results remained stable based on subgroup analyses and interaction tests. However, we observed an inverted U-shaped curve between the two using a smoothed curve fitting in the subgroups of 70 < age ≤ 75 years and females, with inflection points of 6.26 and 6.17, respectively. The results of our study indicate that serum Klotho levels negatively correlate with hypothyroidism among older adults.

## Introduction

With the aging of the population and the extension of life expectancy, the number of elderly people aged 65 and above is increasing, and hypothyroidism has become a common endocrine disease in the elderly^1^. It is estimated that in the UK, hypothyroidism affects nearly 800,000 elderly people annually^2^. A nationwide survey in the United States showed that from 2007 to 2015, the prevalence of hypothyroidism in elderly individuals increased from 5.62 to 8.24%^3^. Research indicates that the prevalence of hypothyroidism in elderly individuals is expected to continue to rise^4^. Therefore, the group of elderly individuals with hypothyroidism requires heightened attention. Compared to younger patients, hypothyroidism in elderly patients presents with more subtle and nonspecific symptoms^5^. If left untreated, it can affect multiple organ systems, including the cardiovascular system and cognitive function^6^. While hypothyroidism in elderly patients causes harm to their physical and mental health, it also poses significant challenges to healthcare systems, encompassing medical care, home care, and public health^4,7^. Therefore, it is necessary to conduct in-depth research on the spectrum of hypothyroidism in elderly individuals and its associated influencing factors, in order to provide a reference basis for guiding the standardized management of hypothyroidism in elderly patients in the future.

The α-Klotho protein (also known as Klotho protein) is a transmembrane protein that acts as a coreceptor for the endocrine fibroblast growth factor 23 (FGF23), which is predominantly expressed in renal tubules and exhibits properties such as anti-aging, antioxidant, and anti-inflammatory effects^8,9^. Decreased serum Klotho levels have been found to be associated with chronic kidney disease^10^, cardiovascular disease^11^, and Alzheimer's disease^12^. Previous studies have also explored the association between Klotho and indicators of thyroid function. For instance, in an animal study conducted by Zhang et al., it was demonstrated that thyroid function indicators increased the levels of Klotho protein in the aortas of rats, thereby inhibiting vascular calcification^13^. Kutluturk et al. found a significant negative correlation between thyroid function indicators and FGF-23 and α-Klotho in obese children and adolescents^14^. Although these studies have revealed potential unexpected connections between Klotho and thyroid function indicators, further analysis of the relationship between Klotho and thyroid dysfunction in specific populations in the real world is lacking. Additionally, there is a lack of research focusing on the association between Klotho and hypothyroidism in the elderly.

Considering the above, our study investigated the relationship between serum Klotho levels and hypothyroidism in older adults aged 65 years and older using the National Health and Nutrition Examination Survey (NHANES) to further elucidate the role of α-Klotho in thyroid function and healthy aging.

## Materials and methods

### Data source and study population

**Figure 1:**
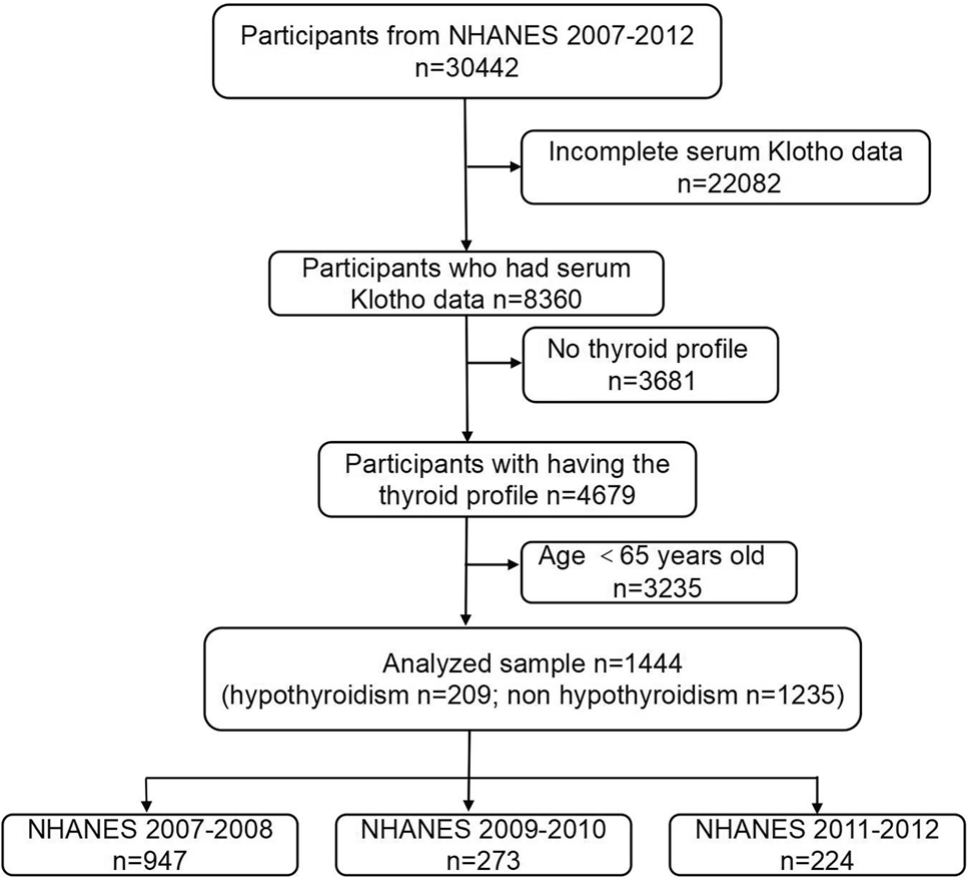
The sample selection flowchart.

The National Health and Nutrition Examination Survey (NHANES) was designed by the National Center for Health Statistics (NCHS) to assess the health and nutritional status of the U.S. deinstitutionalized population using a complex multi-stage cluster probability sampling design^15^. The survey protocol for data collection was authorized by the Ethical Review Board of the National Centre for Health Statistics, and all participants signed an informed consent form^16^. More detailed information about NHANES can be found at http://www.cdc.gov/nchs/nhanes/index.htm. In this study, we analyzed NHANES data from three cycles: 2007–2008, 2009–2010, and 2011–2012, as participants in this module had detailed serum Klotho values and reported thyroid medication use, as well as information on thyroid laboratory tests. The sample selection flowchart is shown in Fig. 1. From the 30,442 participants, we eliminated 22,082 subjects with missing serum Klotho data, 3681 respondents with unreported thyroid test results, and 3235 subjects younger than 65. In the end, 1444 patients in total, including 209 with hypothyroidism and 1235 without, were included in the analysis.

### Serum Klotho

We examined Klotho as an independent variable. Three cycles of serum Klotho samples taken from participants aged 65–79 years, stored at a temperature of − 80 °C, were analyzed in duplicate for experimental data using the IBL ELISA method^20^. Serum Klotho has no specific cut-off point for use as an indicator of biological age and is measured with a sensitivity of 6 pg/mL. A mean of 698.0 pg/mL for serum Klotho levels ranged from 285.8 to 1638.6 pg/mL.

### Measurement of hypothyroidism

Hypothyroidism was the dependent variable in this study. We diagnosed hypothyroidism based on participants' reports of current medications and TSH tests. In accordance with the manufacturer's guidelines, the NHANES document identifies the reference range for normal TSH as 0.34–5.60 mIU/L^21^. Subjects were defined as hypothyroid if they reported that they were currently receiving thyroid hormone replacement therapy (taking levothyroxine or dry thyroid (DT)) regardless of their body's TSH level or if their TSH level was greater than 5.6 mIU/L and they were not taking any antithyroid drugs. Subjects were classified as controls if they did not match the aforementioned requirements. To further characterize the participants, individuals with hypothyroidism were classified based on the degree of the disease (clinical hypothyroidism and subclinical hypothyroidism) according to the FT4 values, using the normal reference range of 0.6–1.6 ng/dL provided in the NHANES documentation^17^. Clinical hypothyroidism is defined as TSH > 5.6 mIU/L, FT4 < 0.6 ng/dL, or the use of levothyroxine^18^. Subclinical hypothyroidism is defined as TSH > 5.6 mIU/L, 0.6 < FT4 < 1.6 ng/dL^19^. In addition, since autoimmune hypothyroidism is caused by autoimmune thyroiditis (AIT)^20^, our study also assessed the association between AIT and serum Klotho levels. AIT is defined as the presence of positive thyroid peroxidase antibodies (TPOAb) or positive thyroglobulin antibodies (TgAb)^21^. According to the normal reference ranges for TPOAb and TgAb provided in the NHANES documentation, TPOAb positivity is defined as ≥ 9.0 IU/mL, and TgAb positivity is defined as ≥ 4.0 IU/mL^17^.

### Covariates

The selection of covariates was based on biological considerations and published literature^22–24^, which include age, sex (categories: male, female), race (categories: non-Hispanic white, others), educational attainment (categories: less than high school, high school, and more than high school), family income-poverty ratio (categories: < 1.0 as low, 1–3 as moderate, and ≥ 3 as high), body mass index (BMI), smoking status (categories: never smoked, ever smoked, and currently smoked), alcohol consumption status ( categories: no alcohol, low to moderate alcohol and heavy alcohol), physical activity (categories: active, not active enough), glomerular filtration rate (eGFR), total cholesterol (TC) concentration in blood samples, diabetes mellitus (categories: yes, no), cardiovascular disease (categories: yes, no) and history of hypertension (categories: yes, no). Of these, never smokers were identified as those who had smoked fewer than 100 cigarettes in their lifetime, ex-smokers were those who had smoked more than 100 cigarettes in their lifetime but no longer smoked, and current smokers were defined as those who had smoked more than 100 cigarettes and smoked either sometimes or continuously. With regard to drinking status, heavy drinkers were defined as men who drink two glasses or more per day and women who drink one glass or more per day, while low- to moderate drinkers were defined as men who drink two glasses less per day and women who consume one glass less per day. Based on the body mass index calculation, BMI was divided into three groups: normal < 25 kg/m^2^, overweight 25–30 kg/m^2^, and obese ≥ 30 kg/m^2^^25^. Glomerular filtration rate (eGFR, ml/min/1.73 m^2^) was assessed using the Chronic Kidney Disease Epidemiological Collaboration equation^26^. Physical activity is calculated based on the duration of moderate and high intensity activities^27^. Cardiovascular disease (CVD) was defined as a patient's self-reported diagnosis of congestive heart failure, coronary artery disease, angina, heart attack, or stroke. More detailed measurement procedures for these variables are on the NHANES website.

### Statistical analysis

Statistical analyses were carried out using R package 4.3.0 (http://www.R-project.org) and EmpowerStats (www.empowerstats.com). Continuous variables were expressed as mean (standard deviation, SD) or median (interquartile range, IQR), while categorical variables were expressed as frequency (percentage). Considering the skewed Klotho distribution, the data were transformed into natural logarithms and expressed in quartiles. When analyzing baseline characteristics, analysis of variance (ANOVA) or non-parametric tests for continuous variables and chi-square tests for categorical variables were used to detect statistical differences between serum Klotho quartiles and between having hypothyroidism or not. Screening for risk factors for hypothyroidism in older people using univariate analysis. Using a logistic generalized linear model, we evaluated whether Klotho correlates with hypothyroidism. Regression models (Models 1 to 2) were tested by adjusting for potential confounders. Model 1 was minimally adjusted for age, sex, and race. Model 2 was fully adjusted for age, sex, race, education, household income to poverty ratio, BMI, smoking status, drinking status, physical activity, diabetes, hypertension, CVD, eGFR, and TC. A smooth curve was fitted to the results, and adjusted odds ratio (OR) and 95% confidence interval (CI) were provided. The association between serum Klotho and hypothyroidism was investigated using a threshold effect analysis model. To assess the stability of the findings, subgroup analyses were performed by stratifying by age, sex, race, BMI, diabetes, CVD, and eGFR. The adjustment strategy was the same as the fully adjusted model (Model 2) except for the variable itself. The log-likelihood ratio test assessed the interaction between serum Klotho levels and subgroup variables. We employed a multivariate logistic regression model to assess the association between serum Klotho levels and clinical hypothyroidism, subclinical hypothyroidism, and autoimmune thyroiditis. The adjustment strategy was consistent with that of Model 1 and Model 2. We employed a multiple imputation (MI) and chained equation technique based on five replications in the R^28^. Multiple imputation was applied to the missing data (drinking status, smoking status, family income-poverty ratio, education level, hypertension, diabetes, BMI) to prevent a deterioration in the effectiveness and bias of the statistical analyses owing to the direct exclusion of missing values. Multivariate logistic regression analysis was performed on the five newly created data sets, and the outcomes were merged. The statistical significance was P < 0.05.

### Ethics approval and consent to participate

The National Center for Health Statistics (NCHS) Research Ethics Review Board approved all study protocols for NHANES. All participants provided written informed consent (https://www.cdc.gov/nchs/nhanes/). Protocol number: #2005–2006 and #2011–2017. We followed all relevant regulations and guidelines when performing all methods.

## Results

### Baseline characteristics of participants

There were a total of 1444 participants, 49.03 per cent of whom were female, with a mean age of 71.38 ± 4.20 years. The mean serum Klotho concentration was 827.23 ± 288.99 pg/mL. Non-Hispanic white people comprised the bulk of participants, and 14.47% of participants had hypothyroidism.

Based on Klotho (ln transformation) quartiles, Table 1 shows the clinical characteristics of the participants. Differences in serum Klotho quartiles were statistically significant with respect to sex, drinking status, hypertension, and glomerular filtration rate (P < 0.05). As shown in Table 1, females and participants with higher glomerular filtration rate values were more likely to have higher Klotho levels between the four groups.

**Table 1:** Baseline characteristics of participants.

Variables	Serum Klotho concentration (pg/mL)				P value
	Q1 (pg/mL)	Q2 (pg/mL)	Q3 (pg/mL)	Q4 (pg/mL)	
N	407	370	355	312	
Age (years)	71.66 ± 4.30	71.44 ± 4.23	71.34 ± 4.03	71.01 ± 4.22	0.224
Sex n (%)					0.001
Male	228 (56.02%)	204 (55.14%)	169 (47.61%)	135 (43.27%)	
Female	179 (43.98%)	166 (44.86%)	186 (52.39%)	177 (56.73%)	
Race/ethnicity n (%)					0.209
Non-Hispanic White	238 (58.48%)	212 (57.30%)	206 (58.03%)	160 (51.28%)	
Others	169 (41.52%)	158 (42.70%)	149 (41.97%)	152 (48.72%)	
Education level n (%)					0.398
Below high school	160 (39.31%)	135 (36.49%)	121 (34.08%)	124 (39.74%)	
High school	83 (20.39%)	91 (24.59%)	85 (23.94%)	78 (25.00%)	
Above high school	164 (40.29%)	144 (38.92%)	149 (41.97%)	110 (35.26%)	
Smoking status n (%)					0.180
Never smoker	160 (39.31%)	163 (44.05%)	161 (45.35%)	154 (49.36%)	
Former smoker	196 (48.16%)	164 (44.32%)	152 (42.82%)	118 (37.82%)	
Current smoker	51 (12.53%)	43 (11.62%)	42 (11.83%)	40 (12.82%)	
Drinking status n (%)					0.002
Nondrinker	102 (25.06%)	110 (29.73%)	102 (28.73%)	104 (33.33%)	
Low-to-moderate Drinker	268 (65.85%)	227 (61.35%)	233 (65.63%)	201 (64.42%)	
Heavy drinker	37 (9.09%)	33 (6.46%)	20 (5.63%)	7 (2.24%)	
Family income-poverty ratio n (%)					0.551
Low	80 (19.66%)	65 (17.57%)	60 (16.90%)	61 (19.55%)	
Middle	198 (48.65%)	187 (50.54%)	179 (50.42%)	169 (54.17%)	
High	129 (31.70%)	118 (31.89%)	116 (32.68%)	82 (26.28%)	
Physical activity n (%)					0.256
Insufficiently active	322 (79.12%)	271 (73.24%)	275 (77.46%)	242(77.56%)	
Active	85 (20.88%)	99 (26.76%)	80 (22.54%)	70 (22.44%)	
Hypertension n (%)	253 (62.16%)	211 (57.03%)	233 (65.63%)	209 (66.99%)	0.031
Diabetes n (%)	93 (22.85%)	81 (21.89%)	80 (22.54%)	86 (27.56%)	0.254
CVD n (%)	100 (24.57%)	94 (25.41%)	85 (23.94%)	78 (25.00%)	0.973
BMI (kg/m^2^)	29.35 ± 5.79	28.64 ± 5.64	29.28 ± 5.80	29.03 ± 5.70	0.293
eGFR (ml/min/1.73 m^2^)	68.90 ± 18.99	71.36 ± 16.74	74.47 ± 16.62	75.74 ± 18.02	< 0.001
TC (mg/dL)	193.93 ± 46.04	192.69 ± 43.39	195.00 ± 42.95	192.44 ± 40.83	0.856

The mean ± SD or median (interquartile range) was calculated for continuous variables, and the frequencies (percentages) were calculated for categorical variables.

CVD: cardiovascular disease; BMI: body mass index; eGFR: glomerular filtration rate; TC: total cholesterol.

The participants were grouped according to the presence or absence of hypothyroidism, and the demographic and health characteristics of the two groups are shown in Supplementary Table 1. Age, sex, race, serum Klotho level, and glomerular filtration rate were statistically significant in determining the presence or absence of hypothyroidism (P < 0.05). Patients with hypothyroidism tend to be female and have lower glomerular filtration rate values as well as lower levels of serum Klotho concentrations than those with non-hypothyroidism.

### Univariate analysis

We performed a univariate analysis of hypothyroidism (Supplementary Table 2). Compared with men, women had a 1.64-fold increased risk of hypothyroidism, a statistically significant difference (P < 0.001). See Supplementary Table 1 for other results.

### Association between Klotho and hypothyroidism

**Figure 2:**
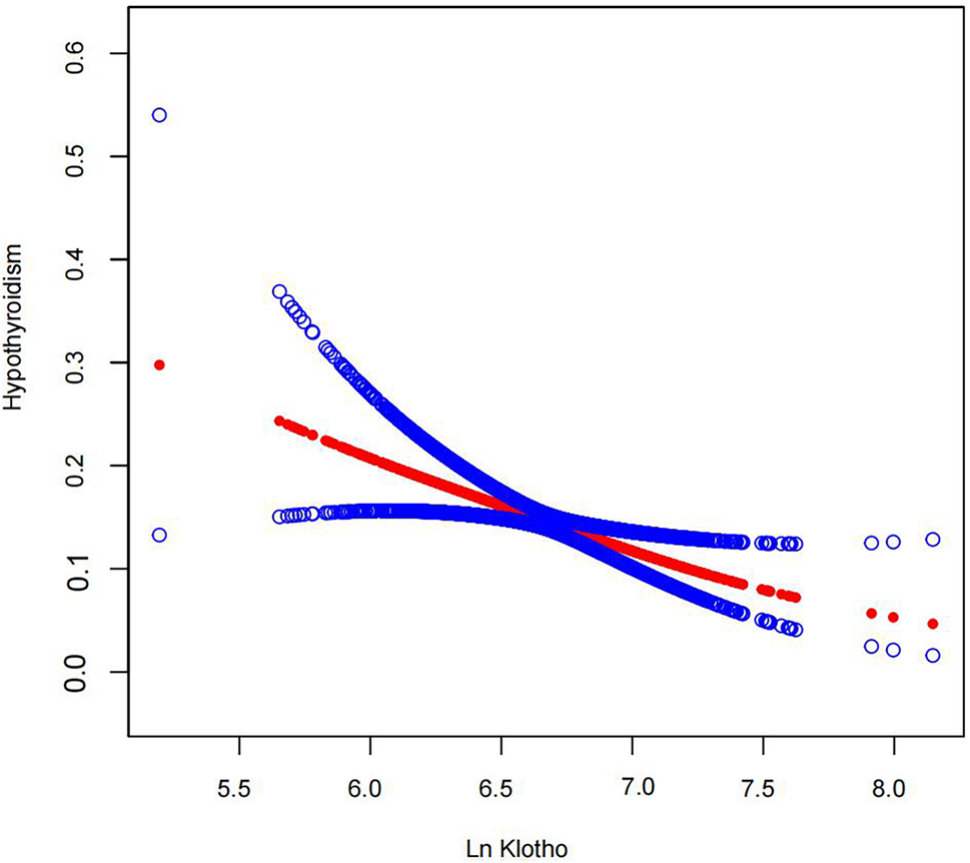
The association between serum Klotho (ln transformation) levels and hypothyroidism.

We used a multivariate logistic regression model to investigate the association between serum Klotho (ln transformation) and hypothyroidism (Table 2). In the unadjusted model, each unit increase in serum Klotho was associated with a 47% reduction in the risk of hypothyroidism (OR = 0.53; 95% Cl 0.34–0.84; P = 0.0064). In minimally adjusted model 1 (adjusted for age, sex, and race), each one-unit increase in serum Klotho was associated with a 54% reduction in the risk of hypothyroidism (OR = 0.46; 95% CI 0.29–0.74; P = 0.0014). In fully adjusted model 2 (adjusted for all covariates), each unit increase in serum Klotho was associated with a 51% reduction in the risk of hypothyroidism (OR = 0.49; 95% CI 0.31–0.80; P = 0.0039). These results suggested that higher serum Klotho levels were associated with a lower risk of developing hypothyroidism, i.e., the two had a negative correlation. In addition, a linear relationship between Klotho (ln transformation) and hypothyroidism was revealed using smoothed curve fitting and threshold effect analysis (Fig. 2 and Supplementary Table 3). When serum Klotho (ln transformation) was divided into quartiles, compared to the first quartile, we found no correlation between serum Klotho quartile 2, quartile 3, and hypothyroidism. Still, the correlation was significant at the highest level. All model results for quartile 4 were OR = 0.52 (95% CI 0.33–0.82, P = 0.0052), OR = 0.48 (95% CI 0.30–0.76, P = 0.0019), and OR = 0.50 (95% CI 0.31–0.81, P = 0.0044), respectively. Additionally, we utilized a multivariable logistic regression model to investigate the association between serum Klotho levels and the degree of hypothyroidism (clinical/subclinical), as well as autoimmune thyroiditis (Supplementary Table 4). After ln transformation, Klotho showed a significant negative correlation with the prevalence of clinical hypothyroidism. In Model 2, compared to the first quartile, ln Klotho in the Q2 group was independently associated with autoimmune thyroiditis (OR = 1.49; 95% CI 1.05–2.12). There was no significant association between serum Klotho levels and subclinical hypothyroidism.

**Table 2:** Association between Klotho (ln transformation) and hypothyroidism.

Exposure	Non-adjusted model	Minimally-adjusted model (Model 1)	Fully-adjusted model (Model 2)
	OR (95% CI) P-value	OR (95% CI) P-value	OR (95% CI) P-value
Ln Klotho	0.53 (0.34, 0.84) 0.0064	0.46 (0.29, 0.74) 0.0014	0.49 (0.31, 0.80) 0.0039
Klotho (quartiles)			
Q1	Reference	Reference	Reference
Q2	0.93 (0.64, 1.36) 0.7054	0.93 (0.63, 1.37) 0.6978	0.92 (0.62, 1.37) 0.6703
Q3	0.82 (0.55, 1.22) 0.3285	0.75 (0.50, 1.12) 0.1641	0.76 (0.51, 1.15) 0.1961
Q4	0.52 (0.33, 0.82) 0.0052	0.48 (0.30, 0.76) 0.0019	0.50 (0.31, 0.81) 0.0044
P for trend	0.0062	0.0015	0.0041

Considering that Klotho is a skewed distribution, the Klotho concentration was transferred by the ln transformation. Model 1 has made minimal adjustments based on age, sex, and race. Model 2 completely adjusted for age, sex, race, education level, family income to poverty ratio, body mass index, smoking status, drinking status, physical activity, diabetes, hypertension, cardiovascular disease, glomerular filtration rate and serum total cholesterol.

### Subgroup analysis

**Figure 3:**
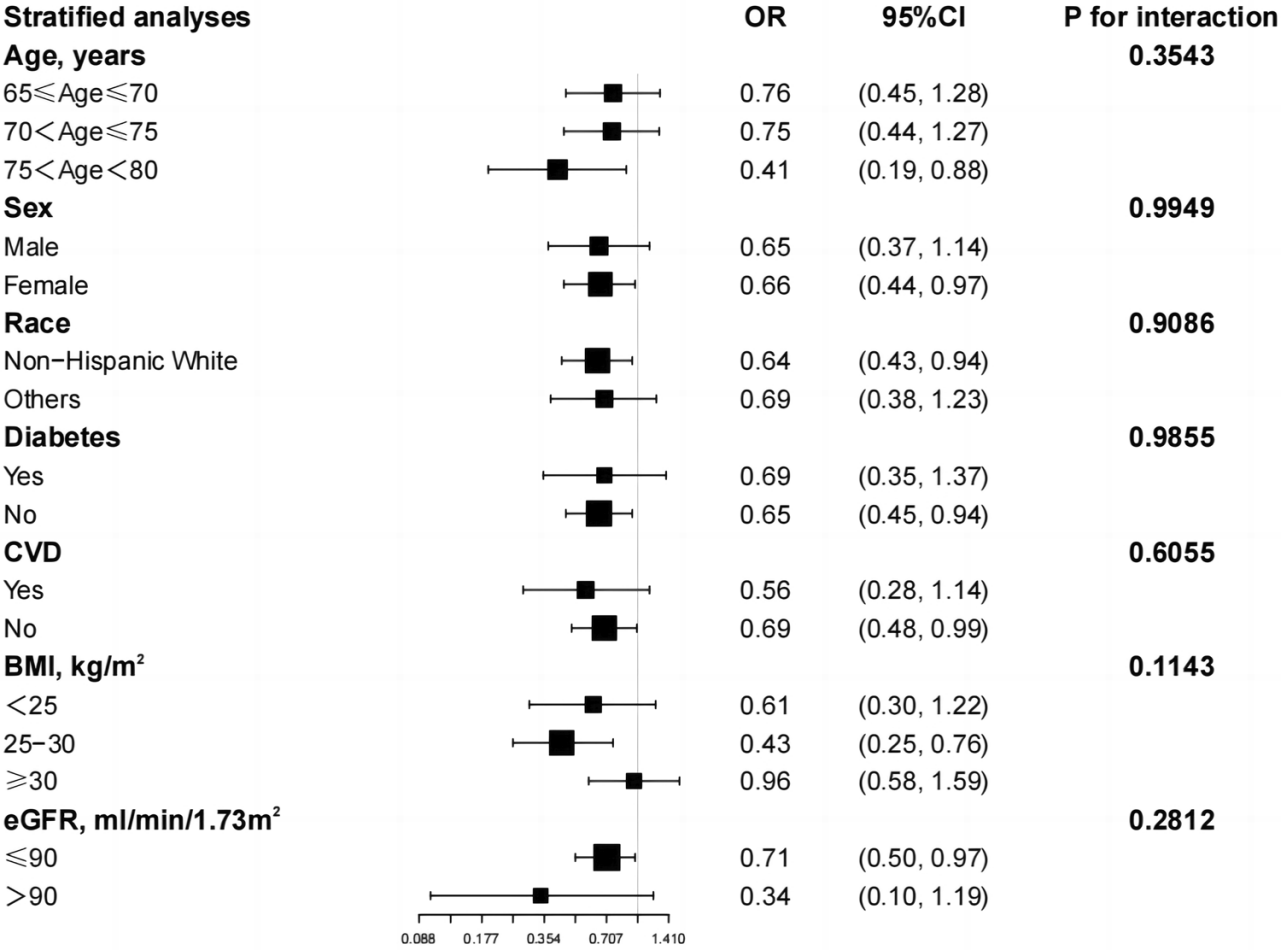
Subgroup analysis for the association between ln Klotho and hypothyroidism.

The findings were tested for stability using subgroup analyses (Fig. 3) and interaction tests, but no interactions were observed. The following subgroups showed significant associations between serum Klotho (ln transformation) levels and hypothyroidism: 75 < age < 80 years (OR = 0.41, 95% CI 0.19–0.88), female (OR = 0.66, 95% CI 0.44–0.97), non-Hispanic White (OR = 0.64, 95% CI 0.43–0.94), no diabetes (OR = 0.65, 95% CI 0.45–0.94), no cardiovascular disease (OR = 0.69, 95% CI 0.48–0.99), 25 < BMI < 30 (OR = 0.43, 95% CI 0.25–0.76), eGFR ≤ 90 (ml/min/1.73m^2^) (OR = 0.71, 95% CI 0.50–0.97). In addition, stratified analyses that would be based on age and sex were analyzed using smoothed curve fitting (Supplementary Figs. 1 and 2). Inverted U-shaped curves were observed in 70 < age ≤ 75 years and in women. Correspondingly, threshold effect analyses (Table 3) showed a significant inverted U-shaped relationship between serum Klotho (ln transformation) levels and hypothyroidism in the subgroups of 70 < age ≤ 75 years and females (P < 0.05 for log-likelihood ratio test). In the 70 < age ≤ 75 subgroup, the ln Klotho inflection point was 6.26. In females, the ln Klotho inflection point was 6.17. On the left side of the inflection point, serum Klotho (ln transformation) levels were positively correlated with hypothyroidism. Conversely, the two had a negative correlation on the right side of the inflection point.

**Table 3:** Threshold effect analysis of serum Klotho (ln transformation) on hypothyroidism using piecewise linear regression.

Outcome	OR (95% CI), P-value
70 < Age ≤ 75	
Inflection point	6.26
Ln Klotho < 6.26	4414.03 (0.52, inf.*), 0.0690
Ln Klotho > 6.26	0.37 (0.12, 1.07), 0.0666
P for the log-likelihood ratio test	0.011
Women	
Inflection point	6.17
Ln Klotho < 6.17	18,670.95 (1.40, inf.*), 0.0424
Ln Klotho > 6.17	0.30 (0.15, 0.61), 0.0008
P for the log-likelihood ratio test	0.003

*The model failed because of the small sample size.

## Discussion

Using the NHANES (2007–2012) database, we analyzed data from 1444 participants aged 65 and over. As far as we know, this is the first cross-sectional study to investigate the relationship between serum Klotho levels and hypothyroidism in a population-based, nationally representative sample of older adults. With confounders adjusted, serum Klotho levels were negatively associated with hypothyroidism prevalence among older adults, and this was consistent across most subgroups. It implies that higher serum Klotho levels may have a prominent role in reducing the risk of hypothyroidism in older adults. We also detected a possible inverted U-shaped relationship in the subgroups of 70 < age ≤ 75 years and females, with inflection points of 6.26 and 6.17, respectively.

One plausible explanation for the association between serum Klotho levels and hypothyroidism in the elderly is aging. During the physiological aging process of the thyroid gland, significant changes occur in the hypothalamic-pituitary-thyroid axis and its hormones^29^. Therefore, although thyroid function can be preserved in the elderly, it may become less stable^30^. On the one hand, under aging conditions, changes in tissue structure gradually accumulate towards malignant transformation, leading to increased vascular permeability and extravasation^31^. These changes slow down the transport efficiency of thyroid hormones in the vascular bed and affect their ability to pass through the endothelial barrier^32^. On the other hand, the aging process results in a decrease in the production of thyroid hormones^33,34^. Overall, aging is closely associated with hypothyroidism in elderly individuals. Klotho is known as an anti-aging gene, and the expression of the Klotho gene decreases with age^35,36^. Moreover, many studies on Klotho in elderly-related diseases indicate that a decrease in serum Klotho levels may contribute to the prevalence of certain chronic conditions in the elderly population^37–39^. Therefore, it is speculated that a decrease in Klotho levels is unfavorable for inhibiting aging, and serum Klotho levels are negatively correlated with the prevalence of hypothyroidism in elderly individuals. Additionally, inflammation may provide another possible explanation. Studies have demonstrated that autoimmune factors are common etiological factors in hypothyroidism^40,41^. Alterations in the immune system can lead to chronic inflammation of the thyroid tissue^42^. Inflammation induces oxidative stress by altering hormones and cytokines. Oxidative stress can inhibit the expression of iodinase, leading to decreased levels of thyroid hormones and subsequently causing hypothyroidism^43,44^. It has been reported that Klotho can regulate inflammation by inhibiting TNF-α-induced adhesion molecule expression and NF-kB activation^24,45^. Additionally, research by Zeng et al. suggested that the downregulation of Klotho and the induction of the RIG-I/NF-kB signaling pathway may be involved in age-related inflammation^46^. Thus, a decrease in serum Klotho levels may favor inflammation, affecting the synthesis and metabolism of thyroid hormones. It is noteworthy that the interaction between aging and inflammation may also play a role in this process^47^. However, considering the complexity of the mechanisms involved, further research will be necessary to elucidate this association in the future.

Sex-stratified analyses showed sex differences in serum Klotho levels. Klotho and hypothyroidism were negatively correlated in men, whereas there was an inverted U-shaped relationship in women. Sex differences in Klotho have been reported in animal and human studies, which may be related to the fact that females face more exposure to stress in the social system^48–51^. In addition, it was suggested that considering genetic factors or interactions between Klotho and other hormones (e.g., glucocorticoids and arginine vasopressin) could help explain sex differences in serum Klotho levels^48^. Age-stratified analyses showed an inverted U-shaped relationship between serum Klotho levels and hypothyroidism in the subgroup 70 < age ≤ 75 years, and we speculated that the limited number masked a linear correlation between the two. The discovery of inflection points in the subgroup analyses reminds us that Klotho management needs to be addressed more closely in our future clinical work. In some cases, it is necessary to keep its levels within the appropriate range. More studies are still needed to confirm further and elucidate the gender and age differences in the association between Klotho and hypothyroidism in older people.

One of our study's main strengths is that it is the first to show an association between serum Klotho levels and hypothyroidism in older adults. In addition, the adequate sample size and proper adjustment for covariates increased the credibility and representativeness of the study. Stable conclusions can be drawn in different subgroups through stratified analysis and interaction tests. This study has the following limitations: firstly, due to the retrospective observational study design, it was impossible to determine the causal and temporal associations between serum Klotho levels and hypothyroidism in older adults. Secondly, our results may have been affected by recall bias because some of the variables collected within the study were self-reported by participants, such as smoking status, drinking status, and physical activity. Furthermore, despite our discovery of a negative correlation between serum Klotho levels in elderly individuals and hypothyroidism, it is important to note that hypothyroidism can be classified according to different criteria. The NHANES lacks relevant data on different classifications of hypothyroidism, which may lead to an underestimation of this correlation.

## Conclusion

Our study concluded that serum Klotho levels negatively correlate with hypothyroidism in older adults. For causality to be assessed, future longitudinal studies and clinical trials are required.

## Supplementary Information

Supplementary Information.

## Data Availability

This study's data from the NHANES are publicly available online at https://www.cdc.gov/nchs/nhanes/.

## References

[1] Calsolaro, V. *et al.* Hypothyroidism in the elderly: Who should be treated and how?. *J. Endocr. Soc.* **3**(1), 146–158 (2019). 30607373 10.1210/js.2018-00207 PMC6309133

[2] Ingoe, L. *et al.* Prevalence of treated hypothyroidism in the community: Analysis from general practices in North-East England with implications for the United Kingdom. *Clin. Endocrinol. (Oxf.)* **87**(6), 860–864 (2017). 28782887 10.1111/cen.13440

[3] Lage, M. J., Espaillat, R., Vora, J. & Hepp, Z. Hypothyroidism treatment among older adults: Evidence from a claims database. *Adv. Ther.* **37**(5), 2275–2287 (2020). 32279175 10.1007/s12325-020-01296-z PMC7467444

[4] Duntas, L. H. & Yen, P. M. Diagnosis and treatment of hypothyroidism in the elderly. *Endocrine* **66**(1), 63–69 (2019). 31482381 10.1007/s12020-019-02067-9

[5] Wilson, S. A., Stem, L. A. & Bruehlman, R. D. Hypothyroidism: Diagnosis and treatment. *Am. Fam. Physician* **103**(10), 605–613 (2021). 33983002

[6] Leng, O. & Razvi, S. Hypothyroidism in the older population. *Thyroid Res.* **12**, 2 (2019). 30774717 10.1186/s13044-019-0063-3 PMC6367787

[7] Chaker, L., Bianco, A. C., Jonklaas, J. & Peeters, R. P. Hypothyroidism. *Lancet* **390**(10101), 1550–1562 (2017). 28336049 10.1016/S0140-6736(17)30703-1 PMC6619426

[8] Kuro, O. M. The Klotho proteins in health and disease. *Nat. Rev. Nephrol.* **15**(1), 27–44 (2019). 30455427 10.1038/s41581-018-0078-3

[9] Castillo, R. F. Pathophysiologic implications and therapeutic approach of Klotho in chronic kidney disease: A systematic review. *Lab. Invest.* **103**(7), 100178 (2023). 37207706 10.1016/j.labinv.2023.100178

[10] Li, S. S. *et al.* Upstream and downstream regulators of Klotho expression in chronic kidney disease. *Metabolism* **142**, 155530 (2023). 36868370 10.1016/j.metabol.2023.155530

[11] Xu, J. P. *et al.* Associations between serum soluble alpha-Klotho and the prevalence of specific cardiovascular disease. *Front. Cardiovasc. Med.* **9**, 899307 (2022). 35795366 10.3389/fcvm.2022.899307 PMC9251131

[12] Zhao, Y. *et al.* Klotho overexpression improves amyloid-beta clearance and cognition in the APP/PS1 mouse model of Alzheimer’s disease. *Aging Cell* **19**(10), e13239 (2020). 32964663 10.1111/acel.13239 PMC7576297

[13] Zhang, J., Chang, J. R., Duan, X. H., Yu, Y. R. & Zhang, B. H. Thyroid hormone attenuates vascular calcification induced by vitamin D3 plus nicotine in rats. *Calcif. Tissue Int.* **96**(1), 80–87 (2015). 25416842 10.1007/s00223-014-9934-8

[14] Kutluturk, Y., Akinci, A., Ozerol, I. H. & Yologlu, S. The relationship between serum FGF-23 concentration and insulin resistance, prediabetes and dyslipidemia in obese children and adolescents. *J. Pediatr. Endocrinol. Metab.* **32**(7), 707–714 (2019). 31211688 10.1515/jpem-2018-0507

[15] Taylor, C. L. *et al.* Critical data at the crossroads: The National Health and Nutrition Examination Survey faces growing challenges. *Am. J. Clin. Nutr.* **117**(5), 847–858 (2023). 36907514 10.1016/j.ajcnut.2023.03.007 PMC10316367

[16] Casagrande, S. S., Lee, C., Stoeckel, L. E., Menke, A. & Cowie, C. C. Cognitive function among older adults with diabetes and prediabetes, NHANES 2011–2014. *Diabetes Res. Clin. Pract.* **178**, 108939 (2021). 34229005 10.1016/j.diabres.2021.108939 PMC8429258

[17] NHANES: NHANES 2009–2010 Laboratory Methods. In*.*

[18] Thavaraputta, S., Dennis, J. A., Laoveeravat, P., Nugent, K. & Rivas, A. M. Hypothyroidism and its association with sleep apnea among adults in the United States: NHANES 2007–2008. *J. Clin. Endocrinol. Metab.* **104**(11), 4990–4997 (2019). 31305928 10.1210/jc.2019-01132

[19] Zhao, G., Wang, Z., Ji, J. & Cui, R. Effect of coffee consumption on thyroid function: NHANES 2007–2012 and Mendelian randomization. *Front. Endocrinol. (Lausanne)* **14**, 1188547 (2023). 37351106 10.3389/fendo.2023.1188547 PMC10282749

[20] Vargas-Uricoechea, H. Molecular mechanisms in autoimmune thyroid disease. *Cells*. **12**(6), 918 (2023). 36980259 10.3390/cells12060918 PMC10047067

[21] Liu, S., Lu, C., He, L., Shan, Z., Teng, W., Li, Y., Liu, T. Vitamin E intake and prevalence rates of thyroid dysfunction and autoimmune thyroiditis: A cross-sectional analysis of NHANES data. *Thyroid*. (2024). 10.1089/thy.2023.0561 38534308

[22] Cai, J. *et al.* Association between serum Klotho concentration and heart failure in adults, a cross-sectional study from NHANES 2007–2016. *Int. J. Cardiol.* **370**, 236–243 (2023). 36351541 10.1016/j.ijcard.2022.11.010

[23] Farias-Basulto, A. *et al.* Circulating levels of soluble Klotho and fibroblast growth factor 23 in diabetic patients and its association with early nephropathy. *Arch. Med. Res.* **49**(7), 451–455 (2018). 30718148 10.1016/j.arcmed.2019.01.008

[24] Zhang, Y. *et al.* Sex differences in the association between serum alpha-Klotho and depression in middle-aged and elderly individuals: A cross-sectional study from NHANES 2007–2016. *J. Affect. Disord.* **337**, 186–194 (2023). 37236270 10.1016/j.jad.2023.05.073

[25] Mahemuti, N. *et al.* Association between systemic immunity-inflammation index and hyperlipidemia: A population-based study from the NHANES (2015–2020). *Nutrients*. **15**(5), 1177 (2023). 36904176 10.3390/nu15051177 PMC10004774

[26] Levey, A. S. *et al.* A new equation to estimate glomerular filtration rate. *Ann. Intern. Med.* **150**(9), 604–612 (2009). 19414839 10.7326/0003-4819-150-9-200905050-00006 PMC2763564

[27] Zhao, G. *et al.* Leisure-time aerobic physical activity, muscle-strengthening activity and mortality risks among US adults: The NHANES linked mortality study. *Br. J. Sports Med.* **48**(3), 244–249 (2014). 24096895 10.1136/bjsports-2013-092731 PMC10938340

[28] Jaddoe, V. W. *et al.* First trimester fetal growth restriction and cardiovascular risk factors in school age children: Population based cohort study. *BMJ* **348**, g14 (2014). 24458585 10.1136/bmj.g14 PMC3901421

[29] Borzi, A. M., Biondi, A., Basile, F. & Vacante, M. Diagnosis and treatment of hypothyroidism in old people: A new old challenge. *Wien Klin. Wochenschr.* **132**(5–6), 161–167 (2020). 31773270 10.1007/s00508-019-01579-8

[30] Mammen, J. S., McGready, J., Ladenson, P. W. & Simonsick, E. M. Unstable thyroid function in older adults is caused by alterations in both thyroid and pituitary physiology and is associated with increased mortality. *Thyroid* **27**(11), 1370–1377 (2017). 28854871 10.1089/thy.2017.0211 PMC5672620

[31] Ungvari, Z., Tarantini, S., Donato, A. J., Galvan, V. & Csiszar, A. Mechanisms of vascular aging. *Circ. Res.* **123**(7), 849–867 (2018). 30355080 10.1161/CIRCRESAHA.118.311378 PMC6248882

[32] Majnarić, L. T., Bosnić, Z., Štefanić, M. & Wittlinger, T. Cross-talk between the cytokine IL-37 and thyroid hormones in modulating chronic inflammation associated with target organ damage in age-related metabolic and vascular conditions. *Int. J. Mol. Sci.* **23**(12), 6456 (2022). 35742902 10.3390/ijms23126456 PMC9224418

[33] Bowers, J. *et al.* Thyroid hormone signaling and homeostasis during aging. *Endocr. Rev.* **34**(4), 556–589 (2013). 23696256 10.1210/er.2012-1056

[34] Calsolaro, V. *et al.* Overt and subclinical hypothyroidism in the elderly: When to treat?. *Front. Endocrinol. (Lausanne)* **10**, 177 (2019). 30967841 10.3389/fendo.2019.00177 PMC6438852

[35] Xu, Y. & Sun, Z. Molecular basis of Klotho: From gene to function in aging. *Endocr. Rev.* **36**(2), 174–193 (2015). 25695404 10.1210/er.2013-1079 PMC4399270

[36] Abraham, C. R. & Li, A. Aging-suppressor Klotho: Prospects in diagnostics and therapeutics. *Ageing Res. Rev.* **82**, 101766 (2022). 36283617 10.1016/j.arr.2022.101766

[37] Paroni, G. *et al.* Klotho at the edge of Alzheimer’s disease and senile depression. *Mol. Neurobiol.* **56**(3), 1908–1920 (2019). 29978424 10.1007/s12035-018-1200-z

[38] Orces, C. H. The association between serum soluble klotho levels and abdominal aorta calcification in older adults. *Aging Clin. Exp. Res.* **34**(6), 1447–1452 (2022). 35091971 10.1007/s40520-021-02053-0

[39] An, C., Chen, X. & Zheng, D. Association between anemia and serum Klotho in middle-aged and older adults. *BMC Nephrol.* **24**(1), 38 (2023). 36797683 10.1186/s12882-023-03081-w PMC9933285

[40] Bogusławska, J., Godlewska, M., Gajda, E. & Piekiełko-Witkowska, A. Cellular and molecular basis of thyroid autoimmunity. *Eur. Thyroid J*. **11**(1), e210024 (2022). 34981746 10.1530/ETJ-21-0024 PMC9142813

[41] Zhang, M. *et al.* Age-specific association between thyroid autoimmunity and hypothyroidism in Chinese adults aged over 65 years: A cross-sectional study. *Front. Endocrinol. (Lausanne)* **14**, 1216308 (2023). 37564984 10.3389/fendo.2023.1216308 PMC10410462

[42] Ferrari, S. M. *et al.* Precision medicine in autoimmune thyroiditis and hypothyroidism. *Front. Pharmacol.* **12**, 750380 (2021). 34867359 10.3389/fphar.2021.750380 PMC8635786

[43] Mancini, A. *et al.* Thyroid hormones, oxidative stress, and inflammation. *Mediators Inflamm.* **2016**, 6757154 (2016). 27051079 10.1155/2016/6757154 PMC4802023

[44] Wang, Y., Sun, Y., Yang, B., Wang, Q. & Kuang, H. The management and metabolic characterization: Hyperthyroidism and hypothyroidism. *Neuropeptides* **97**, 102308 (2023). 36455479 10.1016/j.npep.2022.102308

[45] Valino-Rivas, L. *et al.* Growth differentiation factor-15 preserves Klotho expression in acute kidney injury and kidney fibrosis. *Kidney Int.* **101**(6), 1200–1215 (2022). 35337892 10.1016/j.kint.2022.02.028

[46] Zeng, Y., Wang, P. H., Zhang, M. & Du, J. R. Aging-related renal injury and inflammation are associated with downregulation of Klotho and induction of RIG-I/NF-kappaB signaling pathway in senescence-accelerated mice. *Aging Clin. Exp. Res.* **28**(1), 69–76 (2016). 25986237 10.1007/s40520-015-0371-y

[47] Li, X. *et al.* Inflammation and aging: Signaling pathways and intervention therapies. *Signal Transduct. Target Ther.* **8**(1), 239 (2023). 37291105 10.1038/s41392-023-01502-8 PMC10248351

[48] Behringer, V., Stevens, J. M. G., Deschner, T., Sonnweber, R. & Hohmann, G. Aging and sex affect soluble alpha klotho levels in bonobos and chimpanzees. *Front. Zool.* **15**, 35 (2018). 30250491 10.1186/s12983-018-0282-9 PMC6146871

[49] Guarnotta, V. *et al.* Gender-specific soluble alpha-klotho levels as marker of GH deficiency in children: A case–control study. *J. Endocrinol. Invest.* **45**(6), 1247–1254 (2022). 35279809 10.1007/s40618-022-01757-y PMC9098545

[50] Prather, A. A. *et al.* Longevity factor klotho and chronic psychological stress. *Transl. Psychiatry* **5**(6), e585 (2015). 26080320 10.1038/tp.2015.81 PMC4490291

[51] Rincon-Cortes, M., Herman, J. P., Lupien, S., Maguire, J. & Shansky, R. M. Stress: Influence of sex, reproductive status and gender. *Neurobiol. Stress* **10**, 100155 (2019). 30949564 10.1016/j.ynstr.2019.100155 PMC6430637

