# Supplementary material for: Association between serum Klotho levels and hypothyroidism in older adults: NHANES 2007–2012
Source: Sci Rep. 2024 May 20;14:11477. doi: 10.1038/s41598-024-62297-4 (PMC11106061; doi:10.1038/s41598-024-62297-4)
Supplement: Supplementary file 1 — Supplementary Information. [file 41598_2024_62297_MOESM1_ESM.pdf]

Supplementary Figure 1. The association between ln Klotho and hypothyroidism stratified by age.

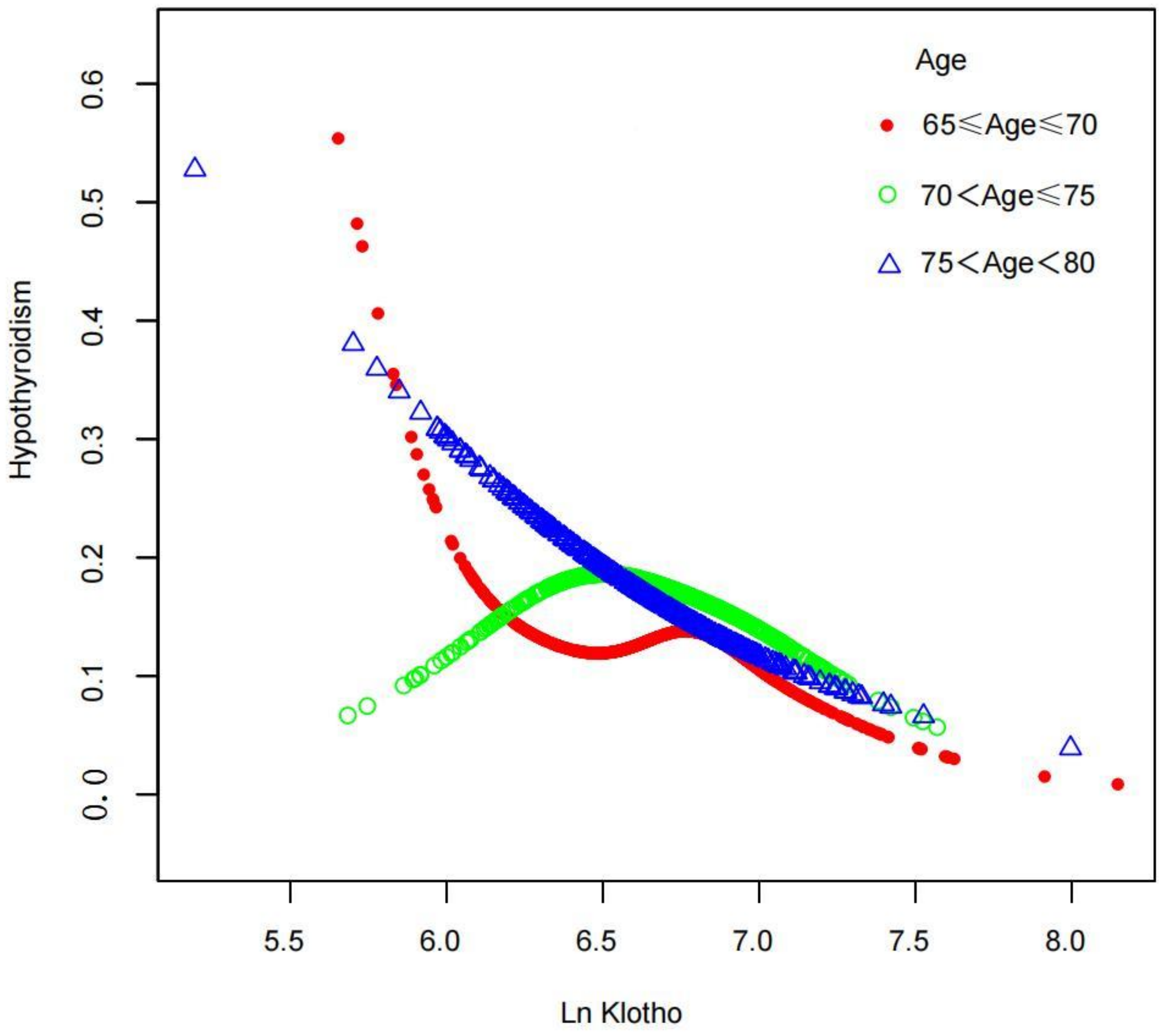

Supplementary Figure 2. The association between ln Klotho and hypothyroidism stratified by sex.

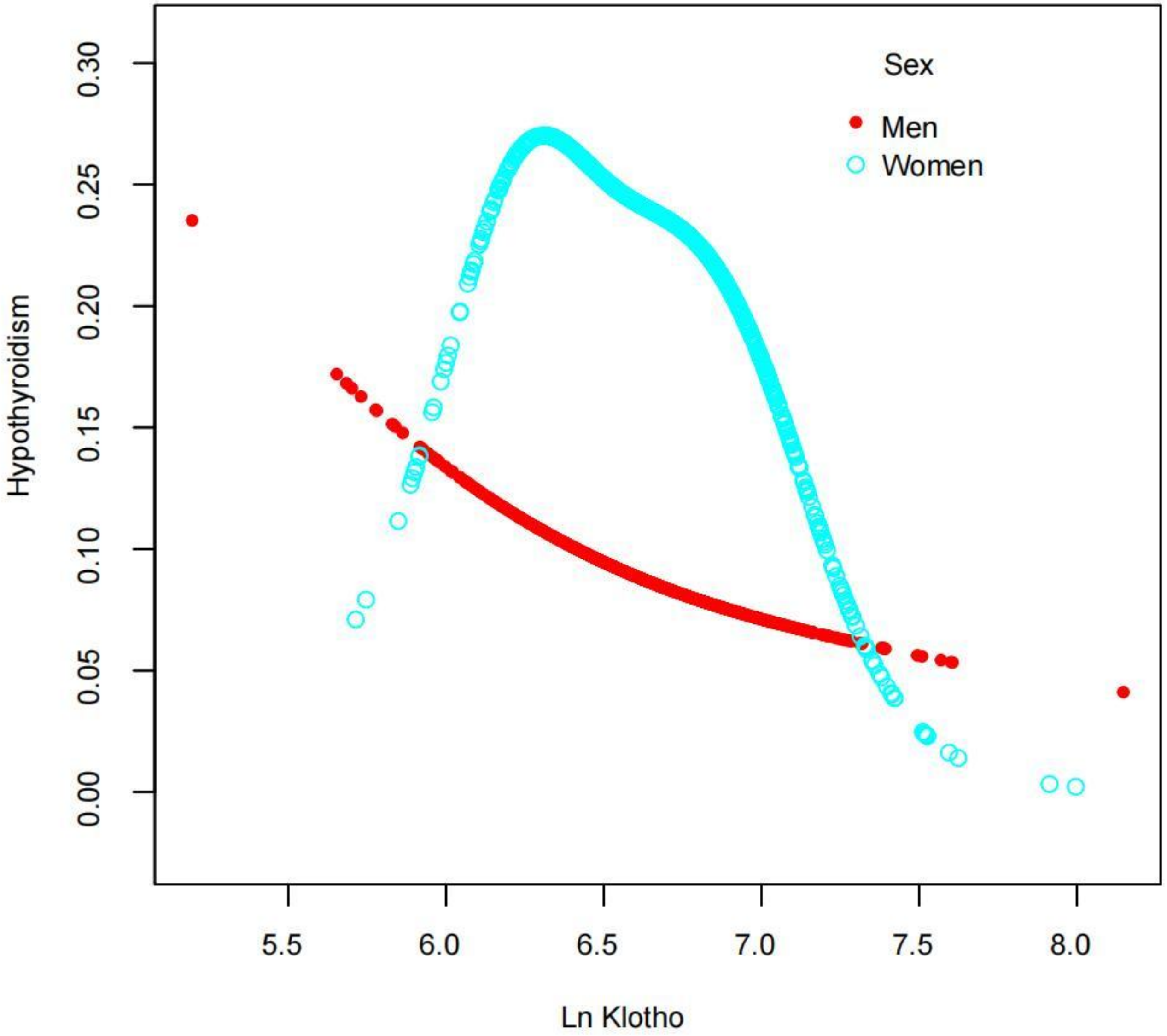

Supplementary Table1. Baseline characteristics of participants.

Variables	Non-hypothyroidism	Hypothyroidism	P -value
N	1235	209	
Age (years)	71.29 ±4.21	71.95±4.15	0.031
Sex n (%)			<0.001
Male	671(54.33%)	65 (31.10%)	
Female	564(45.67%)	144 (68.90%)	
Race/ethnicity n (%)			<0.001
Non-Hispanic White	670 (54.25%)	146 (69.86%)	
Others	565 (45.75%)	63 (30.14%)	
Education level n (%)			0.335
Below high school	471 (38.14%)	69 (33.01%)	
High school	287 (23.24%)	50 (23.92%)	
Above high school	477 (38.62%)	90 (43.06%)	
Smoking status n (%)			0.665
Never smoker	540 (43.72%)	98 (46.89%)	
Former smoker	542 (43.89%)	88 (42.11%)	
Current smoker	153 (12.39%)	23 (11.00%)	
Drinking status n (%)			0.108
Nondrinker	346 (28.02%)	72 (34.45%)	
Low-to-moderate Drinker	808 (65.43%)	121 (57.89%)	
Heavy drinker	81 (6.56%)	16 (7.66%)	
Family Income-poverty ratio n (%)			0.915
Low	228 (18.46%)	38 (18.18%)	
Middle	629 (50.93%)	104 (49.76%)	
High	378 (30.61%)	67 (32.06%)	
Physical activity (%)			0.553
Insufficiently active	946 (76.60%)	164 (78.47%)	
Active	289 (23.40%)	45 (21.53%)	
Hypertension n (%)	772 (62.51%)	134 (64.11%)	0.657
Diabetes n (%)	291 (23.56%)	49 (23.44%)	0.553
CVD n (%)	306 (24.78%)	51(24.40%)	0.907
BMI (kg/m^2)	29.00±5.69	29.59±6.01	0.171
Klotho (pg/mL)	836.34±296.66	773.37±232.07	0.004
eGFR (ml/min/1.73m^2)	72.92±18.05	69.17±16.15	0.001
TC (mg/dL)	193.65±43.98	193.01±40.51	0.845

For continuous variables, mean ± SD or median (interquartile range). For categorical variables, frequencies (percentages).

Abbreviations: CVD: cardiovascular disease; BMI: body mass index; eGFR: glomerular filtration rate; TC: total cholesterol.

Supplementary Table 2. Univariate analysis for hypothyroidism.

	Statistics	OR (95%CI)	P-value
Age (years)	71.38 ±4.20	1.04(1.00,1.07)	0.0349
Sex n (%)			
Male	736 (50.97%)	Reference	
Female	708 (49.03%)	2.64(1.93,3.61)	< 0.001
Race/ethnicity n (%)			
Non-Hispanic White	816 (56.51%)	Reference	
Others	628 (43.49%)	0.51(0.37,0.70)	< 0.001
Education level n (%)			
Below high school	540 (37.40%)	Reference	
High school	337 (23.34%)	1.19 (0.80,1.76)	0.3868
Above high school	567 (39.27%)	1.29 (0.92,1.81)	0.1428
Smoking status n (%)			
Never smoker	638 (44.18%)	Reference	
Former smoker	630 (43.63%)	0.89 (0.66,1.22)	0.4837
Current smoker	176 (12.19%)	0.83 (0.51,1.35)	0.4497
Drinking status n (%)			
Nondrinker	418 (28.95%)	Reference	
Low-to-moderate Drinker	929 (64.34%)	0.72 (0.52,0.99)	0.0424
Heavy drinker	97 (6.72%)	0.95 (0.52,1.72)	0.8634
Family Income-poverty ratio n (%)			
Low	266 (18.42%)	Reference	
Middle	733 (50.76%)	0.99 (0.66,1.48)	0.9689
High	445 (30.82%)	1.06 (0.69,1.64)	0.7793
Physical activity (%)			
Insufficiently active	1110 (76.87%)	Reference	
Active	334 (23.13%)	0.90 (0.63,1.28)	0.5534
Hypertension n (%)			
Yes	906 (62.74%)	Reference	
No	538 (37.26%)	0.93 (0.69,1.27)	0.6573
Diabetes n (%)			
Yes	340 (23.55%)	Reference	
No	1065 (73.75%)	0.99 (0.70,1.40)	0.9490
CVD n (%)			
Yes	357 (24.72%)	0.98 (0.70,1.38)	0.9074
No	1087 (75.28%)	Reference	
BMI (kg/m^2)	29.08±5.74	1.02 (0.99,1.04)	0.1707
eGFR (ml/min/1.73m^2)	72.38±17.84	0.99 (0.98,1.00)	0.0052
TC (mg/dL)	193.55±43.48	1.00 (1.00,1.00)	0.8450
Ln Klotho	6.66±0.33	0.53 (0.34,0.84)	0.0064

Mean ± SD or median (interquartile range) for continuous variables and as frequencies (percentages) for categorical variables.

Abbreviations: CVD: cardiovascular disease; BMI: body mass index; eGFR: glomerular filtration rate; TC: total cholesterol.

Supplementary Table 3. Threshold effects analysis between serum Klotho levels (ln transformation) and hypothyroidism.

Outcome	OR (95%CI), P-value
Fitting by the standard linear model	0.49 (0.31, 0.80) 0.0039
Fitting by the two-piecewise linear model	
Inflection point	6.89
＜6.89	0.68 (0.36, 1.28) 0.2287
＞6.89	0.12 (0.02, 0.85) 0.0334
Predicted at inflection point	-1.78 (-2.04, -1.53)
P for the log-likelihood ratio test	0.113

The adjustment strategy is the same as the fully-adjusted model.

Abbreviations: CI: Confidence Interval; OR: Odds Ratio.

Supplementary Table 4. Multiple logistic regression analysis for the associations between serum Klotho level (ln conversion) and clinical hypothyroidism, subclinical hypothyroidism, and autoimmune thyroiditis.

Exposure	Ln Klotho	Ln Klotho (quartiles)			
		Q1	Q2	Q3	Q4
**Clinical hypothyroidism**					
Non-adjusted model	**0.55 (0.34,0.88)**	Reference	0.94 (0.63,1.41)	0.89 (0.59,1.34)	**0.51 (0.31,0.83)**
Minimally-adjusted model (Model 1)	**0.47 (0.28,0.78)**	Reference	0.92 (0.60,1.40)	0.79 (0.52,1.22)	**0.44 (0.27,0.74)**
Fully-adjusted model (Model 2)	**0.50 (0.30,0.83)**	Reference	0.91 (0.59,1.40)	0.80 (0.51,1.23)	**0.46 (0.28,0.78)**
**Subclinical hypothyroidism**					
Non-adjusted model	2.21 (0.71,6.87)	Reference	1.02 (0.41,2.54)	2.25 (0.70,7.24)	1.67 (0.56,4.94)
Minimally-adjusted model (Model 1)	2.45 (0.74,8.10)	Reference	0.99 (0.39,2.53)	2.57 (0.78,8.45)	1.64 (0.54,4.97)
Fully-adjusted model (Model 2)	2.38 (0.69,8.22)	Reference	1.03 (0.39,2.71)	2.50 (0.75,8.34)	1.54 (0.50,4.74)
**Autoimmune thyroiditis**					
Non-adjusted model	1.14 (0.76, 1.72)	Reference	**1.41 (0.98, 2.04)**	1.25 (0.85, 1.82)	1.09 (0.73, 1.63)
Minimally-adjusted model (Model 1)	1.07 (0.70, 1.63)	Reference	**1.44 (1.00, 2.10)**	1.20 (0.82, 1.78)	1.02 (0.67, 1.54)
Fully-adjusted model (Model 2)	1.12 (0.73, 1.72)	Reference	**1.49 (1.05, 2.12)**	1.21 (0.82, 1.79)	1.06 (0.70, 1.61)

Considering that Klotho is a skewed distribution, the Klotho concentration was transferred by the ln transformation. Model 1 has made minimal adjustments based on age, sex, and race. Model 2 was fully adjusted for age, sex, race, education level, family income to poverty ratio, body mass index, smoking status, drinking status, physical activity, diabetes, hypertension, cardiovascular disease, glomerular filtration rate, and serum total cholesterol.

Supplementary Table 5. Association between Klotho (ln transformation) and hypothyroidism observed using raw data (non-multiple imputation).

Exposure	Non-adjusted model	Minimally-adjusted Model (Model 1)	Fully-adjusted model (Model 2)
	OR (95%CI) P-value	OR (95%CI) P-value	OR (95%CI) P-value
Ln Klotho	0.53(0.34,0.84) 0.0064	0.46(0.29,0.74) 0.0014	0.42(0.25,0.71) 0.0012
Klotho (quartiles)			
Q1	Reference	Reference	Reference
Q2	0.93(0.64,1.36) 0.7054	0.93(0.63,1.37) 0.6978	0.88(0.57,1.35) 0.5480
Q3	0.82(0.55,1.22) 0.3285	0.75(0.50,1.12) 0.1641	0.75(0.48,1.16) 0.1947
Q4	0.52(0.33,0.82) 0.0052	0.48(0.30,0.76) 0.0019	0.37(0.22,0.64) 0.0004
P for trend	0.0062	0.0015	0.0006

Considering that Klotho is a skewed distribution, the Klotho concentration was transferred by the ln transformation. Model 1 has made minimal adjustments based on age, sex, and race. Model 2 completely adjusted for age, sex, race, education level, family income to poverty ratio, body mass index, smoking status, drinking status, physical activity, diabetes, hypertension, cardiovascular disease, glomerular filtration rate and serum total cholesterol.

Abbreviations: OR: odds ratio; CI: confidence interval.

Supplementary Table 6. Distributions of missing data variables (raw data compared to the data after multiple imputation).

Variables	Number with missing data	Raw data	Data after MI	P-value
**Smoking status**	1			1.000
Never smoker		637 (44.14%)	638 (44.18%)	
Former smoker		630 (43.66%)	630 (43.63%)	
Current smoker		176 (12.20%)	176 (12.19%)	
**Drinking status**	81			0.876
Nondrinker		386 (28.32%)	418 (28.95%)	
Low-to-moderate Drinker		880 (64.56%)	929 (64.34%)	
Heavy drinker		97 (7.12%)	97 (6.72%)	
**Education level**	2			1.000
Below high school		539 (37.38%)	540 (37.40%)	
High school		337 (23.37%)	337 (23.34%)	
Above high school		566 (39.25%)	567 (39.27%)	
**Family Income-poverty ratio**	127			0.972
Low		238 (18.07%)	266 (18.42%)	
Middle		672 (51.03%)	733 (50.76%)	
High		407 (30.90%)	445 (30.82%)	
**Hypertension**	2			0.992
Yes		905 (62.76%)	906 (62.74%)	
No		537 (37.24%)	538 (37.26%)	
**Diabetes**	2			1.000
Yes		340 (23.58%)	340 (23.55%)	
No		1063 (73.72%)	1065 (73.75%)	
**BMI (kg/m^2)**	29			0.997
< 25		341 (24.10%)	347 (24.03%)	
25-30		544 (38.45%)	554 (38.37%)	
≥30		530 (37.46%)	543 (37.60%)	
